# Supplementary material for: Assessing the Volume of the Head of the Mandibular Condyle Using 3T-MRI—A Preliminary Trial
Source: Dent J (Basel). 2024 Jul 16;12(7):220. doi: 10.3390/dj12070220 (PMC11276145; doi:10.3390/dj12070220)
Supplement: Supplementary file 1 [file dentistry-12-00220-s001.zip › dentistry-2975136-supplementary.pdf]

Supplementary materials:

(a)

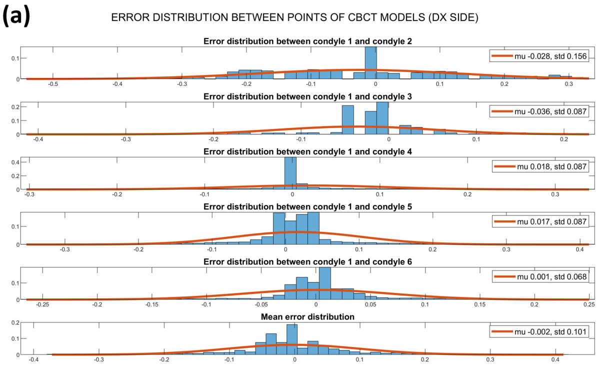

(b)

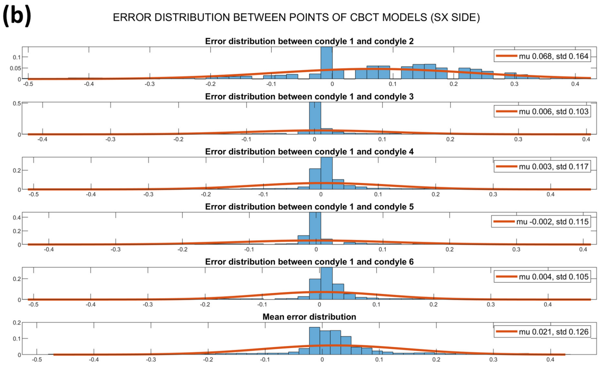

(c)

	MEAN	STANDARD ERROR
GROUP 1	-0.0279 mm	0.0014 mm
GROUP 2	-0.0357 mm	0.0014 mm
GROUP 3	0.0176 mm	0.0014 mm
GROUP 4	0.0170 mm	0.0013 mm
GROUP 5	0.0006 mm	0.0014 mm

GROUP A	GROUP B	P-VALUE
1	2	1.3×10^{-9}
1	3	4.3×10^{-128}
1	4	2.0×10^{-245}
1	5	1.5×10^{-40}
2	3	5.7×10^{-204}
2	4	0
2	5	6.1×10^{-87}
3	4	7.6×10^{-20}
3	5	3.9×10^{-25}
4	5	4.2×10^{-87}

(d)

	MEAN	STANDARD ERROR
GROUP 1	0.0677 mm	0.0017 mm
GROUP 2	0.0056 mm	0.0017 mm
GROUP 3	0.0028 mm	0.0017 mm
GROUP 4	-0.0016 mm	0.0017 mm
GROUP 5	0.0039 mm	0.0017 mm

GROUP A	GROUP B	P-VALUE
1	2	2.2×10^{-253}
1	3	9.6×10^{-253}
1	4	8.7×10^{-157}
1	5	3.6×10^{-82}
2	3	1
2	4	9.5×10^{-13}
2	5	7.5×10^{-47}
3	4	2.2×10^{-12}
3	5	2.9×10^{-46}
4	5	6.4×10^{-12}

Figure S1. Distribution of deviation computed in CloudCompare between CBCT model 1 and the other five for each side. a) and b) Error distribution respectively for right and left side with the mean error distribution plotted in the bottom. c) and d) Tables with mean deviations and standard errors and with resulting p-values of Post Hoc Bonferroni test.

(a)

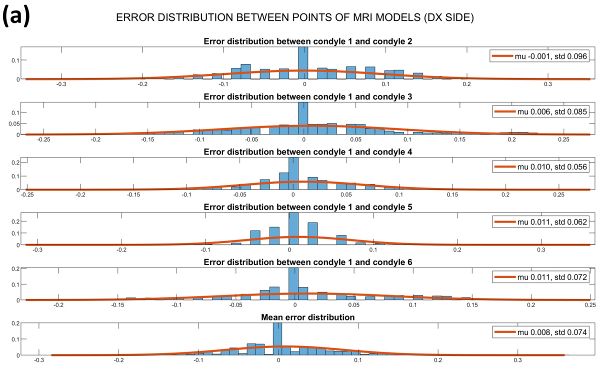

(b)

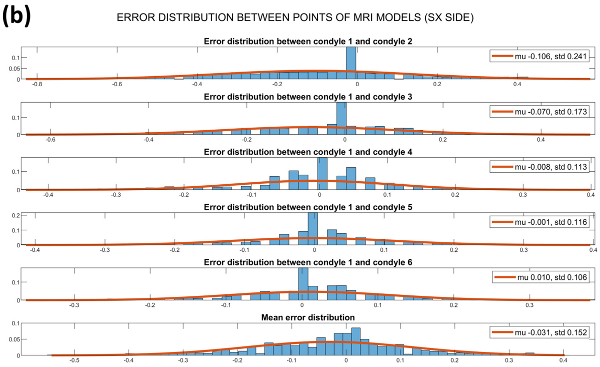

(c)

	MEAN	STANDARD ERROR
GROUP 1	-0.0007 mm	0.0011 mm
GROUP 2	0.0058 mm	0.0011 mm
GROUP 3	0.0104 mm	0.0011 mm
GROUP 4	0.0109 mm	0.0011 mm
GROUP 5	0.0108 mm	0.0011 mm

GROUP A	GROUP B	P-VALUE
1	2	1.4×10^{-1}
1	3	2.0×10^{-10}
1	4	2.6×10^{-15}
1	5	1.2×10^{-4}
2	3	2.3×10^{-4}
2	4	1.1×10^{-7}
2	5	5.6×10^{-1}
3	4	1
3	5	2.0×10^{-1}
4	5	1.4×10^{-3}

(d)

	MEAN	STANDARD ERROR
GROUP 1	-0.1060 mm	0.0022 mm
GROUP 2	-0.0702 mm	0.0022 mm
GROUP 3	-0.0081 mm	0.0022 mm
GROUP 4	-0.0009 mm	0.0022 mm
GROUP 5	0.0097 mm	0.0022 mm

GROUP A	GROUP B	P-VALUE
1	2	9.7×10^{-16}
1	3	1.5×10^{-177}
1	4	1.1×10^{-187}
1	5	7.5×10^{-243}
2	3	6.3×10^{-89}
2	4	2.7×10^{-96}
2	5	1.4×10^{-136}
3	4	1
3	5	9.1×10^{-6}
4	5	5.8×10^{-4}

Figure S2. Distribution of deviation computed in CloudCompare between MRI model 1 and the other five for each side. a) and b) Error distribution respectively for right and left side with the mean error distribution plotted in the bottom. c) and d) Tables with mean deviations and standard errors and with resulting p-values of Post Hoc Bonferroni test.
